# Recent Research Advances in Meat Products

**DOI:** 10.3390/foods10061303

**Published:** 2021-06-07

**Authors:** Mirian Pateiro, Rubén Domínguez, José M. Lorenzo

**Affiliations:** 1 Centro Tecnológico de la Carne de Galicia, Parque Tecnológico de Galicia, rúa Galicia No. 4, San Cibrao das Viñas, 32900 Ourense, Spain; rubendominguez@ceteca.net (R.D.); jmlorenzo@ceteca.net (J.M.L.); 2 Área de Tecnología de los Alimentos, Facultad de Ciencias de Ourense, Universidad de Vigo, 32004 Ourense, Spain

Recent trends in meat products have their origin in the new needs of consumers that are related to nutrition, health concerns, well-being and natural products. Given this situation, the meat sector is forced to develop high-quality and safe meat products. Therefore, novel strategies can be implemented in production, preparation, storage, and distribution systems to induce qualitative and quantitative changes in meat product composition and to optimize the beneficial properties for human health.

Meat and meat products are perishable products that require the use of additives to prevent their rapid deterioration, and to ensure the absence of foodborne microorganisms and pathogenic bacteria [1,2,3]. Lipid oxidation is one of the main causes of quality deterioration in meat and meat products [4]. These reactions lead to a reduction in the nutritional value and sensory quality of meat products. The changes produced during oxidation could condition consumer acceptance (color and texture modification, and appearance of rancid odor and flavor) or even the safety of the product (harmful compounds) [5]. In this regard, antioxidants are added to delay or inhibit the appearance of these undesirable effects [6]. Concern about the possible toxicological effects of synthetic compounds has led to a search for new sources of antioxidant and antimicrobial compounds. Therefore, the use of plant extracts is increasingly demanded by consumers since they are perceived as safe and are Generally Recognized as Safe (GRAS) [7]. Natural extracts could even be introduced in the packaging, through active films that would protect the product from external agents and thus extend its shelf life.

Edible mushrooms could be a potential source of natural antioxidants, since they contain valuable bioactive compounds (proteins, phenolic compounds, and taste enhancers) [8]. In this regard, Novakovic et al. [9] evaluated the possibility of adding *Cantharellus cibarius* as an antioxidant and antimicrobial in cooked sausages. A water decoction was selected as the method of extraction due to the fact that it is simplest and cheapest. Prior to the incorporation of the extract in frankfurters, in vitro antioxidant and antimicrobial activity were tested. Although the decoction limited the extraction of non-polar compounds, the extract showed a high antioxidant potency in a dose-dependent manner. Similar results were also found by other authors [10]. In terms of antimicrobial activity, three Gram-positive (*Staphylococcus aureus*, *Bacillus cereus*, and *Listeria monocytogenes*) and Gram-negative bacterial strains (*Salmonella typhimurium*, *Escherichia coli* (O157:H7), and *Yersinia enterocolitica*), and two yeasts (*Candida albicans* and *Pichia fermentans*) were selected to assess its potential. The results obtained showed a significant antibacterial activity against *Yersinia enterocolitica* (MIC-10 mg/mL) and a slight activity against *Listeria monocytogenes* at the highest concentration (MIC-20 mg/mL) tested, while the fungistatic and fungicidal activity against *Candida albicans* was significantly better. These results are very promising, since the use of *C. cibarius* decoction would allow meat products to be protected against this pathogenic yeast, which could be present during the processing of fresh meat and refrigerated storage, but also in the finished products.

The effects of adding the decoction extract (0.75% and 1.5%) on the physicochemical, microbiological, and sensory quality of frankfurters were evaluated during refrigerated storage. At the beginning of storage, the addition of *C. cibarius* has a trivial influence on the color characteristics of the product, since the total color difference (∆E) values were lower than 3, considered as the limit from which these differences are considered noticeable [11]. These values increased at the end of storage due to the increase in redness (a*), and the decrease in lightness (L*) and yellowness (b*). Regarding texture, no negative effect was observed in frankfurters. Only slight differences were found between treated samples and the control for hardness and chewiness. *C. cibarius* significantly reduced the formation of total aerobic mesophilic bacteria during storage. Finally, frankfurters produced with *C. cibarius* had a partial improvement of odor, taste, and overall quality. The incorporation of *C. cibarius* did not modify, but even improved, the technological and sensory properties of the product, therefore it could be used as a natural ingredient to extend the shelf life of frankfurters during chilled storage.

Ethanol extract of mesquite leaf (EEML) was also suggested by Ramírez-Rojo et al. [12] as a good strategy to prevent lipid oxidation in pork patties. In this regard, a high correlation was found between its polyphenol content (total phenolic content (TPC) of 278.5 mg gallic acid equivalent (GAE)/g, and total flavonoid content (TFC) of 226.8 mg rutin equivalents (RE)/g) and its antioxidant activity (high values for DPPH^●^ radical scavenging activity (FRSA) and reducing power assay (RPA) at 100 µg/mL). Two levels (0.05% and 0.1%) of EEML were added to pork patties, and their effect on physicochemical parameters, lipid oxidation, total antioxidant capacity, and sensory attributes during refrigerated storage were studied. The results were compared to those found in samples without antioxidant and in two positive controls with ascorbic acid and BHT at 0.02%. The addition of EEML did not modify the chemical composition of the patties, while significant differences were found for color and lipid oxidation. Although initially the incorporation of EEML reduced the light pink color of the samples by 27.8% and increased the b* values by 42.1%, in comparison with the control group, at the end of storage patties with EEML displayed the highest a* values. This is in accordance with the values found for metmyoglobin (MMb) in samples with EEML. These values were below the limit that could influence consumers’ purchasing intention (<40% of formation). EEML also improved the oxidative stability of pork patties, even more effectively than potent antioxidants such as BHT, showing the lowest conjugated dienes and TBARS values (40% and 90% of inhibition, respectively). Regarding sensory analysis, there were no significant differences between treatments in color, appearance, odor, flavor, juiciness, fat sensation, and firmness of the cooked pork patties. These results suggest that EEML has great potential as a natural antioxidant in meat products.

As previously mentioned, natural extracts can extend the shelf life of fresh meat and meat products, but they can also improve their health-related characteristics [13,14,15]. In this regard, propolis has been proposed as an ingredient to increase the shelf life of meat products [16]. Previous studies have demonstrated that its extracts have potential health effects, antimicrobial and antioxidant activity. These effects are dependent on the botanical source, season of collection, and phenolic composition [17]. Vargas-Sánchez et al. [18] evaluated the use of propolis extract (PEE) as an antioxidant to improve the oxidative stability of fresh patties during refrigerated storage. The extract was characterized by a high TPC (>400 mg GAE/g), RPA (>50% of reduction), and FRSA (>50% of radical inhibition), with galangin (52.4 mg/g), pinocembrin (44.5 mg/g) and naringenin (27.3 mg/g) the most abundant flavonoids identified. This phenolic content and antioxidant activity were also observed in treated samples (2% of PEE) during storage. Thus, samples treated with PEE showed higher TPC (55.8% and 46.9%) and FRSA (97.8% and 97.9%) than those obtained for control in beef and pork patties, respectively.

At the end of storage, positive effects were observed in color parameters, obtaining the highest a* values in samples treated with PEE (15.0 and 15.7 for beef and pork patties, respectively). In addition, the incorporation of PEE showed the largest decreases in the formation of MMb, followed by samples treated with ascorbic acid and BHT, maintaining the values below the limits that could lead to the rejection of the products by the consumer. The oxidative stability of beef and pork patties increased with the incorporation of PEE, displaying a reduction in lipid (80–88.7% of inhibition) and protein (30.6–47.3% of inhibition) oxidation throughout storage. In the first case, samples treated with antioxidants did not exceed the limit value (higher than 0.6 mg MDA/kg) commonly used as a marker of rancid flavor in meat products [19]. The phenolic compounds identified in PEE, such as quercetin, pinocembrin, kaempferol, and luteolin, would be responsible for the inhibition of the malondialdehyde formation. Similar behavior was observed in protein oxidation, where gallic acid and some flavonols would be the inhibitors of carbonyl compounds. Although the authors did not evaluate the effect of PEE on sensory attributes, it is assumed that the reduction in lipid oxidation would avoid the reduction in sensory qualities associated with changes in color, texture, and appearance of rancid odor and flavor.

*Listeria monocytogenes* is among the pathogenic strains that can put food safety at risk [20]. Raw poultry, processed meat, and ready-to-eat meat products can be contaminated with this microorganism in the slaughterhouse or in different processing steps. Skowron et al. [21] assessed the prevalence and drug susceptibility of *Listeria monocytogenes* from various types of meat (pork, beef, and poultry). The results showed that most *L. monocytogenes* were isolated from poultry, with 32.5% of strains sensitive to all tested antibiotics. Most strains tested were resistant to cotrimoxazole and meropenem, followed by erythromycin, penicillin, and ampicillin. Therefore, it is necessary to use antimicrobials that would allow the microbial load to be reduced or inhibited, thus extending the shelf life of meat products.

The current situation that exists with the COVID-19 crisis makes consumers more worried than ever about their health and well-being [22], which, together with the recommendations of international organizations, is forcing the meat industries to reformulate their products for healthier ones. In this regard, several strategies have been developed to produce low-sodium meat products, despite the fact that NaCl is a widely used preservative in the meat industry [23]. However, it is still necessary to know in detail the effect of salt replacers on specific characteristics (texture, flavor, appearance, moisture, and shelf-life) that are highly valued by the consumers. Vargas-Ramella et al. [24] evaluated the effect of NaCl replacement in the physicochemical quality, volatile and sensorial profile of dry-cured deer cecina. Two salt mixtures were used as NaCl substitutes: mixture I (30% NaCl-70% KCl) and mixture II (30% NaCl-50% KCl-15% CaCl_2_-5% MgCl_2_). This partial replacement had a significant effect on ash and therefore on mineral content (Ca, K, Mg, and Na), coinciding with the composition of each treatment formulation. In this way, mixture II presented higher contents of Mg (104.09 vs. 44.10 mg/100 g) and Ca (265.85 vs. 12.08 mg/100 g) than those observed in mixture I.

Lipid oxidation was also affected by NaCl replacement, with control batch the one that showed the greatest values (3.28 vs. 2.60 and 2.41 mg MDA/kg for control, mixture I and II, respectively). Concerning the volatile compounds, this study confirmed that the generation of volatiles depends on the salt formulations used during the salting stage. Acids (28.72% and 22.95%), followed by hydrocarbons (19.73% and 21.90%) and ketones (19.37% and 20.74%) were the most abundant in control and mixture II treatments, respectively. On the contrary, acids (28.12%), ketones (19.80%), and hydrocarbons (18.83%) were the most prominent in samples treated with mixture I. Regarding hexanal, considered the greatest indicator of lipid oxidation in dry-cured meat, the results were in disagreement with those found in previous studies. In this case, the replacement of NaCl by KCl did not have a pro-oxidative effect and did not result in increased lipid oxidation and hexanal formation.

In contrast, total amounts of free fatty acids and free amino acids, indicators of the lipolysis and proteolysis processes, were not affected by NaCl replacement. Therefore, in the treated samples these phenomena occurred in a very similar way to what happened with the control. Oleic, palmitic, linoleic, stearic and linolenic were the most important fatty acids; while leucine, phenylalanine, alanine, valine, isoleucine, methionine, cysteine and proline were the free amino acids that displayed significant differences among treatments. Except for proline and valine, higher values were obtained in samples salted with alternative salt mixtures. This suggests that it is very important to establish an adequate NaCl replacement, since some of these free amino acids are related to the formation of volatile compounds that contribute to flavor development in the dry-cured products [25]. Therefore, the sensorial properties of the products could be affected. In fact, cecina manufactured with 100% NaCl and salt mixture I had the highest acceptance scores, not observing differences between the two batches.

Another strategy for the production of healthier meat products is to improve the nutritional quality by reducing saturated fat in meat products [26,27]. Carvalho et al. [28] used turkey meat, affected by white-striped myopathy, to develop low-fat cooked sausages with chitosan (1.5% and 3%). This incorporation of chitosan would lead to obtaining a functional product, since it would increase the proportion of fibers in the diet. The resulted product reflected the antioxidant capacity of chitosan, as well as its emulsifying and water retention capacity. In this way, chitosan improved the oxidative stability since TBARS were below the limits of quantification after processing and 56 storage days. In the case of lipid-derived volatiles, a clear antioxidant activity of chitosan was observed, decreasing the release of aldehydes such as hexanal and nonanal. According to the results found by other authors, the inclusion of chitosan increased hardness, since chitosan would act as a binder, thus promoting the formation of a stronger gel with myosin and trapping water. Finally, although samples treated with chitosan received the lowest scores for all sensory attributes, these treatments did not differ from control sausages.

Along with healthy products, consumers also demand quality products linked to upbringing and natural food, obtained with local breeds, better adapted to the environment, since they associate meat from these animals with high quality products [29]. López-Pedrouso et al. [30] evaluated the physicochemical parameters and sensory profile of three Spanish cattle breeds (Asturiana de los Valles, Retinta and Rubia Gallega) under different livestock production systems and pre-slaughter handling conditions. These studies would open a new way to find out the best strategy for the future development of the meat industry in autochthonous breeds, since it would allow to differentiate these products in the meat market.
